# Ergosterol biosynthesis in *Aspergillus fumigatus:* its relevance as an antifungal target and role in antifungal drug resistance

**DOI:** 10.3389/fmicb.2012.00439

**Published:** 2013-01-10

**Authors:** Laura Alcazar-Fuoli, Emilia Mellado

**Affiliations:** Mycology Reference Laboratory, Centro Nacional de Microbiologia, Instituto de Salud Carlos III, Madrid, Spain

**Keywords:** *Aspergillus*, ergosterol biosynthesis, antifungal drugs, resistance mechanisms, transcriptome

## Abstract

Ergosterol, the major sterol of fungal membranes, is essential for developmental growth and the main target of antifungals that are currently used to treat fatal fungal infections. Emergence of resistance to existing antifungals is a current problem and several secondary resistance mechanisms have been described in *Aspergillus fumigatus* clinical isolates. A full understanding of ergosterol biosynthetic control therefore appears to be essential for improvement of antifungal efficacy and to prevent antifungal resistance. An ergosterol biosynthesis pathway in *A. fumigatus* has been proposed with 14 sterol intermediates resulting in ergosterol and another secondary final compound C-24 ethyl sterol. Transcriptomic analysis of the *A. fumigatus* response to host-imposed stresses or antifungal agents is expanding our understanding of both sterol biosynthesis and the modes of action of antifungal drugs. Ultimately, the identification of new targets for novel drug design, or the study of combinatorial effects of targeting sterol biosynthesis together with other metabolic pathways, is warranted.

## ANTIFUNGALS TARGETING ERGOSTEROL AND STEROL BIOSYNTHESIS

Sterols are neutral lipids of eukaryotic cells, among which ergosterol is the main component of fungal membranes. Ergosterol is involved in numerous biological functions such as membrane fluidity, regulation, activity and distribution of integral membrane proteins, and control of the cell cycle (Bard et al., 1993; Gooday, 1995). The essential role of sterols in maintenance of cell membranes make ergosterol and its biosynthetic pathway essential for fungal growth, and a primary target for most, currently available, antifungal drugs to treat severe human fungal infections. Three main classes of antifungal drugs, namely polyenes, allylamines, and azoles, directly target ergosterol itself, or enzymatic steps of its biosynthetic pathway. The polyene amphotericin B (AMB) has represented, for more than 30 years, the standard antifungal therapy for invasive aspergillosis (IA). The AMB mode of action is still poorly understood. It is widely accepted that AMB kills yeast primarily via channel-mediated membrane permeabilization leading to fungal cell death (Ermishkin et al., 1976). However, recent research suggests that AMB kills yeast by simply binding ergosterol so that the channel formation represents a second complementary mechanism that further increases drug potency and the rate of cell death (Gray et al., 2012). The allylamine group of antimycotics interferes at the early-stage of ergosterol biosynthesis by inhibiting the enzyme squalene epoxidase (Erg1). Although squalene epoxidases from various origins have been investigated with respect to substrate requirements, cofactors, and inhibitors, no structural model is available; and the domains responsible for enzymatic activity and inhibitor interactions are not well understood (Ruckenstuhl et al., 2007). The cidal action of allylamines is closely associated with the development of high intracellular squalene concentrations, which are believed to interfere with fungal membrane function and cell wall synthesis (Ryder, 1992). Allylamines are not used to treat IA, however potential use in combination with azoles, polyenes, or echinocandins in the management of severe drug-resistant or refractory mycoses has been proposed (Krishnan-Natesan, 2009). Finally, the main category of antifungal agents used against *Aspergillus* is azole drugs, such as voriconazole (VCZ) and itraconazole, and a new generation of azoles that includes posaconazole, isavuconazole, and albaconazole. Azoles revolutionized medical mycology due to their broad spectrum and reduced toxicity compared to AMB. Azoles block the ergosterol biosynthesis pathway via inhibition of 14-α sterol demethylase (Cyp51/Erg11), a key enzyme that removes the methyl group at position C-14 of precursor sterols. Inhibition of ergosterol synthesis at this biochemical level results in toxic sterol accumulation and cell death (Ghannoum and Rice, 1999).

However, although the use of antifungal drugs has increased survival of patients with invasive fungal disease, the mortality rate associated with these infections remains extremely high. This situation becomes more complicated because emerging resistance to the existing antifungals is a current problem and secondary resistance mechanisms have been described in clinical isolates (Howard and Arendrup, 2011; Shapiro et al., 2011; van der Linden et al., 2011a). Full understanding of the ergosterol biosynthesis pathway in *A. fumigatus* is essential to new antifungal drug design and for improving the activity of existing ones. This review summarizes current knowledge about the enzymatic sequence and regulation of the biochemical reactions involved in ergosterol biosynthesis with a view to understanding the main mechanisms of antifungal resistance to drugs which target ergosterol or its biosynthesis.

## *Aspergillus fumigatus* ERGOSTEROL BIOSYNTHESIS PATHWAY

**FIGURE 1:**
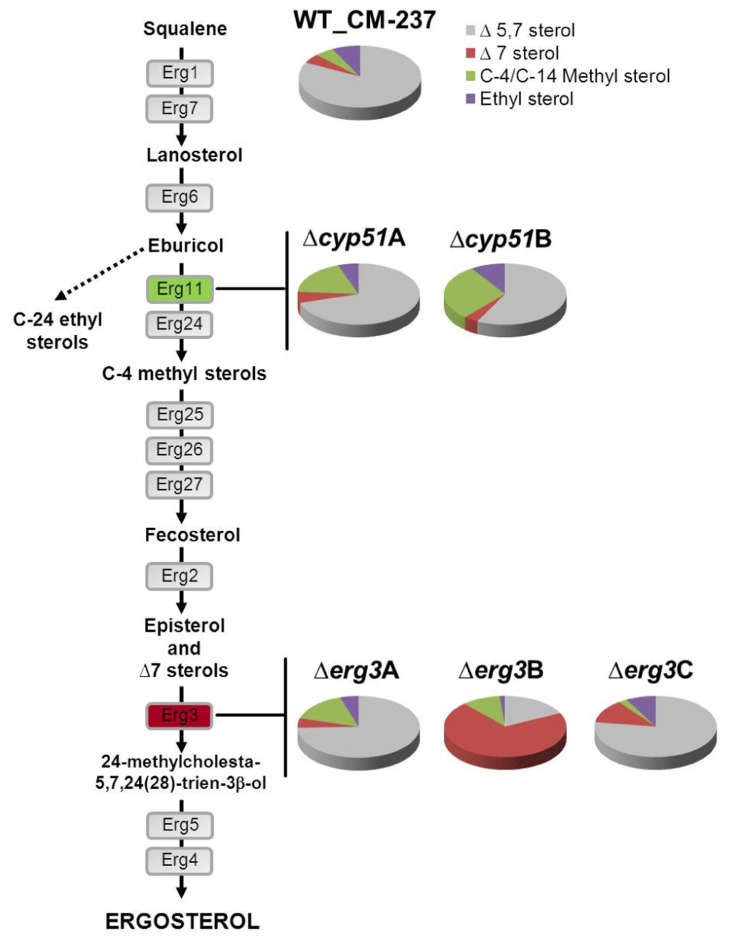
**Ergosterol biosynthetic pathway**. Sterols of *A. fumigatus* wild-type strain (CM-237), and Δ*cyp*51A, Δ*cyp*51B (marked in green), Δ*erg*3A, Δ*erg*3B, and Δ*erg*3C (marked in red) derived mutant strains. Sterols are clustered and represented in cake plots as the percentage of total sterols: Δ5 sterols (1, 2, 3, 4, 6, 8 in gray), C-4/C-14 methyl sterols (11, 12, 13, 14 in green), Δ7 sterols (5, 7, 9 in red), and ethyl sterols (10 in purple) according to the sterol identification in *A. fumigatus* (**Table 1**).

The biosynthesis of ergosterol involves about 20 enzymes and includes the synthesis of squalene from mevalonate (Ferreira et al., 2005; Alcazar-Fuoli et al., 2008). This route is well known in *Saccharomyces cerevisiae* which provides a model for studying this pathway in other eukaryotes (Fryberg et al., 1973). Depletion of ergosterol biosynthetic gene functions is lethal in *S. cerevisiae*, due to complete prevention of ergosterol production. Deviating from the prototypical pathway described in *S. cerevisiae*, other more complex biosynthetic pathways have been proposed in some fungi and also in plants, suggesting that the enzymatic pathways of ergosterol biosynthesis are specific to fungal taxa (Fryberg et al., 1973; Nes et al., 1989). In *A. fumigatus*, the study of this pathway at a genomic level, demonstrated the existence of multiple genes encoding key enzymes such as two distinct 14-α sterol demethylases (Cyp51A and Cyp51B; Mellado et al., 2001) and three C-5 sterol desaturases (Erg3; Alcazar-Fuoli et al., 2006). The classical ergosterol biosynthesis route was reconsidered in *A. fumigatus* based on the existence of multiple genes which putatively encode enzymes with the same or duplicated function in the pathway (**Figure 1**). Genetic and biochemical analysis of azole susceptible wild-type strains and a panel of clinical azole resistant strains identified a total of 14 sterols showing a similar sterol pattern composition, with ergosterol as the main sterol for all of them (**Table 1**). Each of the azole resistant strains had a different antifungal susceptibility pattern that correlated with different mutations in the *cyp*51A gene. The lack of differences between sterols highlights the fact that the resistance mechanism in those strains was target-dependent and not sterol-dependent, as will be discussed later. Instead, the sterol composition of single enzyme defective *A. fumigatus* mutants was rather different depending on the lacking enzyme (Alcazar-Fuoli et al., 2008). Mutants deleted in 14-α sterol demethylases (*cyp*51A and *cyp*51B) had less ergosterol than the parental strain and accumulated C-4, and C-14 methyl sterols; mainly eburicol (**Figure 1**). Accumulation of eburicol, together with a decrease in ergosterol, was much more prominent when the *cyp*51B gene was deleted compared to Δ*cyp*51A strain. Downstream in the pathway, the synthesis of ergosterol involves the transformation of fecosterol, consisting of double-bond rearrangements in the steroid nucleus and in the side chain, isomerization of the double connection in the C-8 to the C-7 followed by the desaturation at C-5 and C-22, and the reduction of the C-24. The enzymatic sequence of these three last steps could differ between fungal taxa and growth conditions (Nes et al., 1989). Single gene deletion of each of the C-5 sterol desaturases (Erg3A, Erg3B, and Erg3C) revealed a different sterol profile in terms of total amount of ergosterol and sterol composition. Again this phenotype was different depending on the deleted enzyme. While the absence of the genes *erg3*A and *erg*3C did not significantly affect total ergosterol biosynthesis, the deletion of *erg*3B caused a dramatic ergosterol decrease (70%) and a vast accumulation of the Δ7 sterols (**Figure 1**). Although the sterol composition revealed differences according to the enzyme that was deleted, none of the single gene deletions, at 14-α sterol demethylase or C-5 sterol desaturase level, was lethal for *A. fumigatus* or influenced its virulence (Mellado et al., 2005; Alcazar-Fuoli et al., 2006). This suggests that although *A. fumigatus* might use one of the enzymes Cyp51 or Erg3 for normal growth, this fungal pathogen is able, including during infection, to adapt and compensate that lack of an enzyme function.

**Table 1:** Relative amount of sterols (% of total sterols) in different *A. fumigatus* wild-type (CM-237), *cyp51* deleted strains (Δ*cyp51*A Δ*cyp51*B), and azole resistance mutants strains with different *cyp51*A point mutations (CM-796:G54V; CM-3269:TRL98H; CM-2159: M220K).

Sterol names	Strains					
	CM-237*	Δ*cyp51*A	Δ*cyp51*B	CM-796*	CM-3269*	CM-2159*
1) 24-methylcholesta-5,7,9(11),22-tetraen-3β-ol	1.09	3.69	2.74	0.85	1.18	0.92
2) 24-methylcholesta-5,8,22-trien-3β-ol	0.91	0.87	0.77	0.90	0.80	0.85
3) 24-methylcholesta-5,7,9,22-tetraen-3β-ol	0.71	5.69	4.57	0.00	0.00	0.00
4) 24-methylcholesta-5,7,22-trien-3β-ol (Ergosterol)	78.12	59.48	48.82	84.08	83.70	88.44
5) 24-methylcholesta-7,22 (28)-dien-3β-ol	2.62	1.84	1.18	1.51	1.67	1.09
6) 24-methylcholesta-5,7,22,24(28)-tetraen-3β-ol	0.48	0.41	0.25	0.32	0.21	0.28
7) 24-methylcholesta-7,22,24(28)-trien-3β-ol	0.66	1.44	0.71	0.82	0.51	0.20
8) 24-methylcholesta-5,7,24(28)-trien-3β-ol	0.91	0.14	0.34	0.08	0.19	0.16
9) 24-methylcholesta-7,24(28)-dien-3β-ol (Episterol)	1.51	2.55	2.01	3.59	1.84	1.08
10) 24-Ethylcholesta-5,7,22-trien-3β-ol	7.48	5.48	9.26	2.43	6.46	5.10
11) 4,4,14-trimethylcholesta-8,24-dien-3β-ol (Lanosterol)	1.06	1.39	2.87	3.63	0.29	0.18
12) 4α,24-dimethylcholesta-8,24(28)-dien-3β-ol	0.99	2.03	0.98	0.73	0.00	0.82
13) 4,4,14,24-tetramethylcholesta-8,24(28)-dien-3β-ol (Eburicol)	1.14	9.54	21.95	0.41	1.30	0.23
14) 4,4,24-trimethylcholesta-8,24(28)-dien-3β-ol	2.04	5.44	3.56	0.65	0.62	0.65

* Geometric mean of MICs to antifungal agents of *A. fumigatus* strains: CM-237: ITC (0.25 μg/ml), VRC (0.48 μg/ml), POS (0.06 μg/ml), FLC (1094.55 μg/ml), AMB (0.26 μg/ml), TRB (5.4 μg/ml); CM-796: ITC (16 μg/ml), VRC (1.19 μg/ml), POS (1.68 μg/ml), FLC (1094.55 μg/ml), AMB (0.15 μg/ml), TRB (2.38 μg/ml; CM-2159: ITC (16 μg/ml), VRC (1.19 μg/ml), POS (2 μg/ml), FLC (1094.55 μg/ml), AMB (0.3 μg/ml), TRB (3.17 μg/ml); CM-3269: ITC (16 μg/ml), VRC (4 μg/ml), POS (0.5 μg/ml), FLC (1094.55 μg/ml), AMB (0.2 μg/ml), TRB (2 μg/ml).

## ANTIFUNGAL RESISTANCE MECHANISMS RELATED TO ERGOSTEROL OR ITS BIOSYNTHETIC PATHWAY

Three of the most widely used antifungal drugs, triazoles, polyenes, and allylamines, are aimed at ergosterol, and they are either fungicidal but toxic to the host (polyenes) or fungi static and more vulnerable to resistance (triazoles). Triazoles are inhibitors that target the ergosterol biosynthetic pathway by binding to the Cyp51 family of cytochrome P450s (14-α sterol demethylases) causing the depletion of ergosterol biosynthesis and the accumulation of lanosterol or eburicol (Kelly et al., 1995). *A. fumigatus* contains two different, but related Cyp51 proteins, encoded by *cyp*51A and *cyp*51B (Mellado et al., 2001) that are functional 14-α sterol demethylases (Martel et al., 2010; Warrilow et al., 2010). The lethality caused by double gene deletion has been shown, although neither gene is itself essential (Mellado et al., 2005; Hu et al., 2007). Moreover, the importance of *A. fumigatus* Cyp51 proteins in sterol biosynthesis and their significance in azole susceptibility have been extensively studied (Alcazar-Fuoli et al., 2008, 2011; Martel et al., 2010). Also, the *in vitro* and *in vivo* correlation of azole resistance has been widely documented; with a clear association of resistant *A. fumigatus* strain isolation and lack of patient response to therapy (van der Linden et al., 2011a). *A. fumigatus* azole resistance can arise either from modification of the Cyp51A target, or by its overexpression. Resistance acquired through exposure to azoles in the patientis correlated with *cyp*51A single mutations (Diaz-Guerra et al., 2003; Mann et al., 2003; Nascimento et al., 2003; Mellado et al., 2004; Howard et al., 2006; Albarrag et al., 2011), while resistance caused by *cyp*51A overexpression is specifically linked to the combination of a single amino acid substitution (a leucine to histidine change, L98H), together with the presence of two tandem copies of a 34-bp sequence in the promoter of the *cyp*51A gene. This latter type of resistance mechanism may have evolved in the environment through the exposure of the fungus to azole fungicides used in agriculture (Snelders et al., 2008). In isolation the tandem 34-bp promoter insertions cannot explain the observed azole cross resistant phenotype (Mellado et al., 2007), however recent work has demonstrated the essentiality of this 34-bp promoter sequence in maintaining the wild-type expression of *cyp*51A, and suggested the presence of other regulatory elements upstream of the tandem insertions (Paul et al., 2012). Further mechanisms of azole resistance include overexpression of drug transporters of the ABC- and/or MFS-type (Tobin et al., 1997; Slaven et al., 2002; Nascimento et al., 2003), however clinically observed resistance appears thus far to be limited to mutations which modify the Cyp51A target site. The sterol content of azole resistant *A. fumigatus* strains bearing different Cyp51A point mutations suggests that their azole resistant phenotypes are not the result of perturbations of the ergosterol biosynthetic pathway (Table 1). It is more likely that azole resistance in these variants results from a reduced affinity of drug binding to the Cyp51A enzyme. In this sense, 3D protein models of Cyp51A in combination with azoles have provided the basis to address how point mutations can affect azole drug resistance (Alcazar-Fuoli et al., 2011; Fraczek et al., 2011).

Regarding the polyene antifungals, the association between *A. fumigatus* susceptibility to AMB *in vitro* and clinical outcome is unclear. Secondary resistance to AMB is generally not observed, even in patients whose therapy has failed (Moosa et al., 2002), yet resistant mutants have been spontaneously induced in the laboratory (Manavathu et al., 1998) and resistance to AMB is well documented for many other *Aspergillus* species (Blum et al., 2008; van der Linden et al., 2011b; Hadrich et al., 2012). Acquired resistance to AMB has been most extensively evaluated in yeasts and is associated with mutations in the *ERG*3 gene, which is linked to qualitative and quantitative alterations of membrane lipids and an absence of ergosterol (Kelly et al., 1997; Morio et al., 2012). However, the deletion of three independent *erg*3-like genes in *A. fumigatus* did not affect susceptibility to AMB, despite marked alterations of sterol composition and a decrease in total ergosterol (Alcazar-Fuoli et al., 2006). Other studies have determined that neither ergosterol content, cell wall composition, or lipid peroxidation levels correlate with heightened *A. terreus* AMB resistance, only that a higher level of catalase production in *A. terreus* might contribute to AMB resistance since it promotes oxidative damage of fungal cell membranes through generation of reactive oxygen species (Blum et al., 2008).

Terbinafine belongs to the allylamine class of antifungals that inhibit squalene epoxidase (Erg1). Erg1 catalyzes the first oxygenation step in sterol biosynthesis and is suggested to be one of the rate-limiting enzymes in this pathway. Alterations in *A. fumigatus* *erg*1 gene dosage can promote terbinafine resistance (Liu et al., 2004), however, the first terbinafine resistance mechanism was described in *A. nidulans* and associated with the activity of salicylate 1-monooxygenase (*sal*A), a well-characterized naphthalene-degrading enzyme, suggesting that resistance could follow degradation of the naphthalene ring contained in terbinafine (Graminha et al., 2004). Also, a point mutation, F391L, in the squalene epoxidase enzyme was found to confer a strong terbinafine resistance phenotype. The equivalent mutation was introduced into the homologous gene of *A. fumigatus* resulting in terbinafine resistance (Rocha et al., 2006). Since terbinafine is not currently used in the management of IA, the appearance of terbinafine-resistant strains in the clinical setting is unlikely, however, its potential use in combination with other antifungal drugs should be considered.

## ALTERNATIVES TO IMPROVE ANTIFUNGAL EFFICACY AND TO MINIMIZE DRUG RESISTANCE

Global gene expression studies identified that ergosterol biosynthesis is likely to be highly sensitive to environmental perturbations. Among them, adaptation to the host environment when *A. fumigatus* initiates disease is particularly interesting. The transcriptome of *A. fumigatus* during early stages of infection in the neutropenic murine lung identified reduced expression of genes encoding ergosterol pathway functions (Erg11, Erg24, and Erg3) relative to laboratory cultures (McDonagh et al., 2008).

Although the target site of azole activity is well studied, the role of other proteins in the mode of action of these drugs in fungi is poorly understood. Recently, a critical role for SrbA-mediated regulation of ergosterol biosynthesis and triazole drug interactions in *A. fumigatus* has been reported (Blosser and Cramer, 2012). SrbA was identified by transcriptional profiling under hypoxia conditions as a regulator of ergosterol biosynthetic genes in response to low oxygen levels. The Δ*srb*A mutant strain was attenuated in virulence and it was hypersusceptible to fluconazole and VCZ (Willger et al., 2008). Also, several genes encoding enzymes that require high levels of oxygen were found to be transcriptionally repressed in the absence of SrbA, including the enzymes Erg6, Erg11, Erg24, Erg25, and Erg3. The relationship between ergosterol regulation and hypoxia was also demonstrated by the analysis of the sterol profile of the Δ*srb*A which showed a decrease in ergosterol levels and accumulation of C-4 methyl sterols, suggesting a blockage of C-4 demethylation. In addition, the apparent control of *cyp*51 transcript levels by SrbA suggests an additional target for drug development. A role for SrbA in the development of triazole resistance has also been suggested, since alterations in this transcription factor or its DNA-binding affinity could transcriptionally initiate changes that could alter target abundance and would lead to triazole resistance (Blosser and Cramer, 2012).

In a similar manner, analysis of sterol composition in iron-depleted and replete cultures revealed decreased ergosterol biosynthesis during iron starvation and the accumulation of two types of sterol intermediates C-4 and Δ7. The genome-wide expression response of *A. fumigatus* in a shift from iron-depleted to iron-replete conditions (10, 30, 60, 120, and 240 min compared with that from 0 min) showed that Erg24 and Erg25 functions were down-regulated for all of the time points, and in contrast Erg11 was up-regulated at 30 and 60 min of iron exposure (Schrettl et al., 2008). In conditions of iron limitation, *A. fumigatus* uses siderophore-assisted iron uptake, which in turn uses mevalonate as a precursor. The results described above, together with the characterization of functional siderophore deleted mutants, probed the link between fungal ergosterol and siderophore biosynthesis in *A. fumigatus* via the compound mevalonate. The latter would appear to feed both biochemical pathways (Yasmin et al., 2012). Also, in hypoxic conditions SrbA was found to activate siderophore-mediated iron uptake in response to hypoxia and iron starvation in part by transcriptional activation of another transcription factor, HapX (Blatzer et al., 2011).

Transcriptional regulation of ergosterol biosynthesis in *A. fumigatus* has also been studied under antifungal exposure in laboratory cultures. Microarray analysis indicated down-regulation of the *erg*6 gene under both AMB and VCZ exposure (da Silva Ferreira et al., 2006; Gautam et al., 2008). Erg6 has a role in the synthesis of secondary sterols (ethyl sterols), and its down-regulation suggests that *A. fumigatus* alters the synthesis of secondary sterols in response to AMB or VCZ. However, different results were found for the *A. fumigatus* Cyp51-encoding genes, which were both down-regulated under VCZ treatment and up-regulated with AMB. These results may indicate the existence of differential responses for overcoming the distinct stresses imposed by these drugs. In addition, genes encoding for Erg24, Erg25, and Erg3 were differentially expressed when *A. fumigatus* cells were cultured in the presence of VCZ (da Silva Ferreira et al., 2006).

Collectively, the above observations suggest that ergosterol biosynthesis is prone to perturbation by environmental conditions, and in a manner dependent upon Erg6, Erg11, Erg24, Erg25, and Erg3 functions, thereby highlighting these gene functions as alternative or synergic inhibitors of the pathway. An important finding in *A. fumigatus* was the identification of C-24 ethyl sterols. These sterols can also be final metabolites of the sterol pathway and they are predominantly found in higher plants, though absent in mammalian cells, which cannot alkylate the C-24 of sterols. In plants C-24 ethyl sterols have multiple roles to play in growth and development, however, few reports exist on the detection of 24-ethyl sterols in fungi, and their role in *A. fumigatus* remains unknown. C-24 alkylation is catalyzed by *S*-adenosyl-methionine-sterol-C-methyltransferases (SAMs). In *S. cerevisiae* the methylation is catalyzed by *erg6* which converts zymosterol into fecosterol, and at least two methyltransferases are involved in consecutive methylation reactions leading to 24-ethyl sterols in higher plants (Bouvier-Nave et al., 1998). In *A. fumigatus*, only one Erg6 has been identified that could transform lanosterol to eburicol and may also be involved in the production of C-24 ethyl sterols. The sterol profile analysis of *A. fumigatus* strains showed that the synthesis of 24-Ethylcholesta-5,7,22-trien-3β-ol is sensitive to pathway perturbations. In a Δ*cyp*51B mutant a lack of ergosterol is compensated by an increase of 24-Ethylcholesta-5,7,22-trien-3β-ol, probably because the activity of the pathway becomes diverted toward the synthesis of alkyl sterols. However, when *erg*3B is suppressed, ergosterol levels are decreased together with 24-ethylcholesta-5,7,22-trien-3β-ol, confirming that this enzyme would also be involved in the desaturation of C-5,7 alkyl sterols.

Genome wide studies are providing us with a very useful platform from which to dissect the linkages between cellular sterol biosynthesis and other cell functions or metabolic pathways. Such new findings might be further explored for novel drug development or for possible combinatorial therapeutic strategies to fight invasive fungal diseases. Among them, genes encoding enzymes involved in cellular stress, cell wall synthesis, and transport have been found to be differentially expressed under antifungal exposure or underhost imposed stresses. To conclude, *A. fumigatus* is able to synthesize ergosterol, even with suppression of several enzymes in the pathway. Biosynthetic control under different environmental circumstances is observed, demonstrating that *Aspergillus* has alternatives to overcome severe drawbacks. Therefore, the precise knowledge of this complex biosynthetic pathway could facilitate the future development of novel and more selective antifungal drugs in order to improve efficacy and to minimize drug resistance.

## Conflict of Interest Statement

The authors declare that the research was conducted in the absence of any commercial or financial relationships that could be construed as a potential conflict of interest.
